# Left ventricular hypertrophy among chronic kidney disease patients in Ghana

**DOI:** 10.11604/pamj.2017.28.79.9183

**Published:** 2017-09-27

**Authors:** Yaw Ampem Amoako, Dennis Odai Laryea, George Bedu-Addo, Bernard Cudjoe Nkum, Jacob Plange-Rhule

**Affiliations:** 1 Renal Medicine and Dialysis Unit, Department of Medicine, Komfo Anokye Teaching Hospital, Kumasi, Ghana; 2 Disease Control Department, Ghana Health Service, Accra, Ghana; 3 School of Medical Sciences, Kwame Nkrumah University of Science and Technology, Kumasi, Ghana

**Keywords:** Left ventricular hypertrophy, chronic kidney disease, Ghana, sokolow-lyon criteria, prevalence

## Abstract

**Introduction:**

The presence of left ventricular hypertrophy (LVH) in patients with Chronic Kidney Disease (CKD) is associated with worsening cardiovascular outcomes. There is a dearth of data on LVH in Ghanaian CKD patients.

**Methods:**

This was a cross sectional study carried out at the Komfo Anokye Teaching Hospital (KATH) in Kumasi, Ghana. A questionnaire was used to obtain information on clinical features of CKD. The MDRD-4 equation was used to calculate eGFR. Information on the prevalence and factors associated with electrocardiographic left ventricular hypertrophy were obtained during the initial assessment.

**Results:**

About 64.5% of the 203 participants were male and the mean age was 43.9 ± 17.8 years. Most subjects (79.8%) had stage 5 disease. The mean systolic and diastolic blood pressures were 167.86 ± 39.87 and 101.8 ± 24.4 respectively. Approximately 43% of respondents had LVH. eGFR correlated negatively with LVH. High systolic pressure (OR 4.9, CI 2.4 – 10.4; p < 0.05), high diastolic pressure (OR 8.1, CI 4.0 – 16.1; p < 0.05) increased pulse pressure (OR 3.4 CI 2.6-9.3, p < 0.05), increased body mass index (OR 3.6 CI 1.7-11.2, p < 0.001) as well as male gender (OR 4.7, 95% CI 2.4 – 9.1; p <0.05) were associated with the presence of LVH.

**Conclusion:**

LVH is common in our cohort. High pulse pressure, high DBP, increased BMI and male gender are significant associated factors. Adequate treatment of high blood pressure as well as early detection of LVH and interventions aimed at prevention and/or regression of LVH are to be encouraged.

## Introduction

Chronic Kidney Disease (CKD) is a worldwide public health challenge with adverse outcomes of kidney failure, cardiovascular disease and premature death [1]. CKD patients are at significantly increased risk for both morbidity and mortality from cardiovascular disease [2, 3]. In fact cardiovascular disease (CVD) is the leading cause of death in CKD populations in the developed world [2]. The situation is no different in developing countries like Ghana. Eghan and colleagues [4] reported CVD as responsible for 40% of deaths among a dialysis cohort. Traditional CVD risk factors such as hypertension, diabetes mellitus, smoking, and dyslipidaemia are common in individuals with CKD than those with normal renal function [5–8]. Other non-traditional or uraemia related CVD risk factors-anaemia, C reactive protein, carotid intima -media thickness and asymmetric dimethyl arginine have been identified among patients with CKD [5, 9]. Left ventricular hypertrophy (LVH) has been reported as a prevalent cardiovascular complication among CKD patients. The presence of LVH in CKD patients generally portends a negative prognostic value because it can contribute to the development of heart failure, ischaemic heart disease, arrhythmias and sudden death. LVH is a strong predictor of cardiovascular morbidity and mortality among CKD patients [10]. Several modalities exist for the assessment of LVH. Compared to electrocardiography [11–14], echocardiography [15, 16], computed tomography, and magnetic resonance imaging [17] have been used to provide more accurate assessments of myocardial mass. However, electrocardiograms are more convenient and less expensive compared to the other imaging modalities. Several electrocardiographic left ventricular hypertrophy (ECG LVH) criteria exist. However, the standard voltage criterion reported by Sokolow and Lyon [18] remains the most widely used. LVH determined by this criterion is independently associated with echocardiographic LVH, and cardiovascular morbidity and mortality [19]. Regression of ECG LVH is also associated with reduction in adverse cardiovascular outcomes [20]. Among Ghanaian CKD patients however, the prevalence of this important CVD risk factor is not known. We set out to examine the prevalence and determinants of LVH among CKD patients during their initial assessment at a tertiary hospital adult renal service.

## Methods

This was a cross sectional study carried out over a 1-year period at the Komfo Anokye Teaching Hospital (KATH). KATH is the second largest hospital in Ghana and caters for patients in middle and northern zones of the country. The hospital provides clinical services for both adult and paediatric renal patients. Haemodialysis is the main mode of Renal Replacement Therapy (RRT). Chronic Kidney Disease (CKD) patients attending the renal clinic over a 1 year period were eligible for inclusion in the study. Consecutive patients diagnosed with CKD based on the case definition age 18 years and above were eligible for inclusion. Eligible patients were not to be undergoing dialysis. Patients attending the renal clinic with diagnosis other than CKD and CKD patients who did not provide informed consent were excluded from this study. Informed consent was obtained from all study participants or their legal representatives prior to inclusion in the study. A questionnaire was administered to each patient to obtain the demographic data and clinical history. A detailed clinical examination was performed on all respondents to obtain information including: weight, height, systolic and diastolic blood pressure. The height was measured with the patient standing barefooted on flat surface. Blood pressure was measured using Intellisense™ M3 automatic Blood Pressure monitor (Omron Healthcare Europe BV, Netherlands). The pulse pressure was calculated as the difference between the systolic and diastolic blood pressures. A midstream urine sample was analysed for proteinuria using dipstick testing (DIRUI Industrial Co. Ltd, Changuchun, Jilin 130012 P.R. China) and the degree of proteinuria classified as absent (urine dipstick negative, or present (dipstick positive).

Abdominopelvic ultrasonography was done to assess the architecture of the kidneys. The haemoglobin (Hb) level and Hb indices were measured at KATH haematology laboratory with an auto analyser (Sysmex KS-21N, Sysmex Corp, Japan). Liver function tests (LFT), blood urea nitrogen (BUN) and serum creatinine levels (based on modified Jaffe method) were measured at the KATH biochemistry laboratory using an auto-analyser (BT 3000 PLUS, Biotecnica Instruments S.p.a, Rome, Italy). Estimated glomerular filtration rate (eGFR) was calculated using the Modification of Diet in Renal Disease (MDRD-4) equation and the stage of CKD noted.

**ECG measurement and interpretation:** ECG was measured at a paper speed of 25mm/s, at a gain of 10mm/mV (or 5mm/mV) using an AT 200 ECG machine (Schiller AG, Switzerland). Sokolow-Lyon LVH was defined as sum of S wave in lead V1 and R wave in lead V5 or V6 ≥ 35mm and/ or R wave in lead aVL ≥ 11mm. ECG interpretations were done by a Physician Specialist and verified by another senior Physician.

**Ethical statement:** The study was approved by and conformed to the ethical guidelines of the Committee on Human Research Publications and Ethics (CHRPE) of the School of Medical Sciences, Kwame Nkrumah University of Science and Technology and the Komfo Anokye Teaching Hospital.

**Definition of terms:** 1) CKD was defined as the presence of kidney damage, manifested by abnormal albumin excretion or decreased kidney function, quantified by measured or estimated glomerular filtration rate (GFR) that persists for more than three months. In the absence of previous data on eGFR or markers of kidney damage, chronicity was inferred from clinical presumption of kidney disease for >3 months; 2) Proteinuria was classified as absent (urine dipstick negative), or present (urine dipstick positive); 3) Hypertension was defined as the presence of a persistently elevated systolic blood pressure ≥ 140mmHg and/or diastolic blood pressure ≥ 90mmHg in patients, and/or the use of antihypertensive drugs and/or past medical history of hypertension; 4) Hypertension was noted as the cause of renal disease in cases if medical record indicated hypertension was present before onset of kidney disease and if there was absence of proteinuria, normal renal function indices, and preserved renal sizes in presence of hypertension early in the illness. 5)Anaemia was defined as haemoglobin (Hb) level < 11 g/dL.

**Statistical analysis:** Continuous variables are presented as means ± SDs and as percentages for dichotomous variables. Differences in characteristics in subjects with and without LVH were evaluated using χ^2^ (chi square test). Associations between LVH and parameters were analysed using multivariate logistic regression modeling. Epi Info version 7.1.2.0 was used for analysis and a p value <0.05 was considered statistically significant.

## Results

A total of 203 participants were recruited into the study. The baseline demographic and clinical characteristics of participants are represented in Table 1. The majority of respondents were male (64.5%) giving a sex ratio (M/F) of approximately 1.9: 1. The mean age (± SD) of the respondents was 43.9 ± 17.8 years. The modal age group was 40-49 years and accounted for 25.1% of cases. The mean age for male respondents was 42.6 ± 17.2 and for females 45.5 ± 18.7. There was no statistically significant difference between the mean ages of the two groups (p = 0.114). Patients with LVH had a higher body mass index (27.3 vs 26.3, p=0.04) and were slightly younger than those without LVH. The median serum creatinine was 1325 μmol/L (range 136-3939). Majority of respondents (79.8%) had stage 5 CKD (55.7% vs 24.1% for males and females respectively). The mean systolic and diastolic blood pressures for all study participants were 167.9 ± 39.8 mmHg and 101.8 ± 24.4 mmHg respectively. Patients with left ventricular hypertrophy (LVH) had significantly higher SBP ((OR 4.9 CI 2.4-10.4 p <0.00001), DBP (OR 8.1 CI 4.0-16.1 p<0.0001) and higher pulse pressure (OR 3.4 CI 2.6-9.3, p = 0.0007) than those without LVH (Table 1) .

**Table 1:** Profile of CKD patients with and without LVH

Characteristic	All patients, N (%)	ECG-LVH present	ECG-LVH absent	p value
Number (%)	203	88 (43.3)	115 (56.7)	
Age, mean (±SD)	43.9 ±17.8	43.6 ±17.3	46.5±8.6	0.12
Gender (male), n (%)	131 (64.5)	72 (55.0)	59 (45.0)	< 0.00001
BMI (kg/m2), median (IQR)	26.5(24.7-28.3)	27.3 (25.6-27.7)	26.3(24.8-27.1)	<0.04
Hypertensive*	43 (21.2)	37 (86.0)	6 (14.0)	<0.002
SBP (mmHg)	167.9 ± 39.8	174.2 ± 38.6)	151.8 ± 32.4	< 0.00001
DBP (mmHg)	101.8 ± 24.4	107.8 ± 25.2	92.3 ± 23.9	< 0.0001
Pulse pressure (mmHg)	123.8±37.2	128.3±29.6	117.6±26.5	0.0007
Anaemia	176 (86.7)	69 (78.4)	107 (93.0)	0.38
Proteinuria	176 (86.7)	72 (81.8)	104 (90.4)	0.8
**CKD stage**				
3	29 (14.3)	11 (12.5)	18 (15.7)	<0.05
4	12 (5.9)	5 (5.7)	7 (6.0)	
5	162 (79.8)	72 (81.8)	90 (78.3)	
**Age range**				
< 60 years	167 (82.3)	81 (92.0)	86 (74.8)	0.04
>60 years	36 (17.7)	7 (8.0)	29 (25.2)	

**BMI:** body mass index, **SBP:** systolic blood pressure, **DBP:** diastolic blood pressure

* cases with hypertension as underlying cause of CKD

The prevalence of left ventricular hypertrophy as determined by the Sokolow-Lyon LVH was 43.3%. There was a negative correlation between eGFR and LVH (95% CI -0.38 to -0.13; p = 0.0001). There were more males than females (35.5% versus 7.8%) with LVH (OR 4.7, 95% CI 2.4-9.1; p < 0.05). Ninety-two percent (92%) of patients with LVH were aged < 60 years. Majority (81.8%) of cases with LVH had stage 5 CKD (95% CI 72.9-89.9). CKD stages 3 and 4 accounted for 12.5% and 5.7% of cases with LVH respectively (Table 1).

The abdominopelvic ultrasound results are represented in Table 2. The commonest sonographic finding in our cohort was reduced renal sizes and was present in 38.9% of cases. Thirty three percent of patients had reduced renal sizes with loss of corticomedullary differentiation. Two patients had polycystic kidney disease while 6.9% had normal sonographic findings (Table 2).

**Table 2:** Ultrasonography findings in CKD patients

Sonographic finding	Frequency (%)
Reduced renal sizes only	79 (38.9)
Loss of corticomedullary differentiation (CM)	24 (11.8)
Reduced renal sizes and loss of CM differentiation	67 (33.0)
Increased renal sizes	8 (3.9)
Polycystic kidneys	2 (1.0)
Normal	14 (6.9)
Prostatic enlargement with obstructive uropathy	9 (4.4)

The analysis of association between LVH and risk factors is presented in Table 3. High systolic (OR 4.9 CI 2.4-10.4 p < 0.00001) and diastolic (OR 8.1 CI 4.0-16.1 p<0.0001) blood pressures, pulse pressure (OR 3.4 CI 2.6-9.3, p = 0.0007), and male gender (OR 4.7 CI 2.4-9.1 p < 0.00001) were associated with the presence of left ventricular hypertrophy. Patients with hypertension as the underlying cause of CKD have an increased risk for developing LVH (p <0.002). Employment status (OR 2.5 CI 1.3-4.6, p = 0.02) and high body mass index (OR 3.6 CI 1.7-11.2, p < 0.001) were also associated with the presence of LVH. Anaemia and proteinuria were not associated with the presence of LVH. In a logistic regression modelling, high diastolic blood pressure, high pulse pressure, high BMI and male gender were found to be significantly associated with LVH.

**Table 3:** Risk factors for LVH in CKD patients

Variable	Unadjusted Odds ratio (95% CI)	p value	Adjusted Odds ratio (95% CI)	p value
DBP	8.1 (4.0-16.1)	<0.0001	1.07 (1.0-1.1)	0.05
Employed	2.5 (1.3- 4.6)	0.02	1.67 (0.79-3.5)	0.32
Anaemia	0.7 (0.4- 1.4)	0.38	-	-
SBP	4.9 (2.4- 10.4)	<0.00001	1.0 (0.98- 1.02)	0.06
Pulse pressure	3.4 (2.6- 9.3)	0.0007	1.4 (1.0- 3.1)	0.04
BMI	3.6 (1.7- 11.2)	<0.001	1.9 (1.4- 3.7)	0.04
**Proteinuria**				
Present	1.5 (0.6- 3.8)	0.8	-	-
Absent	1.0			
**Gender**				
Male	4.7 (2.4- 9.1)	<0.00001	2.91 (1.27-6.67)	0.01
Female	1.0			
**Age**				
<60 years	1.3 (1.16- 1.71)	0.04	1.1 (1.02-1.09)	0.47
>60 years	1.0		1.0	

## Discussion

The mean age of study participants was 43.9 ± 17.8 years. This is in agreement with other published studies that found CKD to be commoner in the third to fifth decades of life [4, 21]. There was a preponderance of males (M/F = 1.9:1). This is similar the population studied by Chijioke and colleagues in Nigeria [13]. About 86% of patients had stage 4 or 5 disease. The higher incidence of CKD among males is likely due to the fact that CKD and the risk factors are more prevalent among males than females. CKD patients in sub-Saharan Africa appear to be younger than those in the developed world [21, 22]. Several factors account for the earlier onset of CKD in the developing world. In addition to the high prevalence of infections which predispose to chronic glomerulonephritis, there are few screening programmes to identify populations at risk for CKD while detection and control rates of CKD risk factors such as hypertension and diabetes mellitus are low [23]. CVD risk is known to be highest in advanced or late stages of CKD [2, 8].

There was an inverse relationship between left ventricular hypertrophy and the estimated glomerular filtration rate. Patients with low eGFR (corresponding to more advanced CKD), are more likely to have left ventricular hypertrophy. Complications such as hypertension, anaemia, fluid overload and arterial stiffening are more common in advanced CKD. These factors contribute to the development of LVH, hence the high incidence of LVH observed in the later stages of CKD. Levin and colleagues [24] demonstrated that the prevalence of LVH increased with progressive renal decline: 26.7% of patients with creatinine clearance (Ccr) greater than 50 mL/min had LVH, 30.8% of those with Ccr between 25 and 49 mL/min had LVH, and 45.2% of patients with severe renal impairment (Ccr <25 L/min) had LVH (P = 0.05). In the present study, 37.9% of stage 3 had LVH, 41.7% of stage 4 had LVH, and 44.4% with CKD stage 5 had LVH (p <0.05). This is in agreement with findings from other developing [13, 14, 16] and developed [12, 15, 25, 26] countries.

The overall high prevalence of LVH (43.3%) by Sokolow-Lyon criteria is in agreement with the 45% by Chijioke et al [13] working in Nigeria and the 40% by Stewart et al [12] but contrasts the 83% reported by Nwankwo and colleagues [14]. It is also higher than the 8% prevalence from the study by Agarwal and colleagues [11] from the United States. It must be noted however that the study by Agarwal et al excluded patients with stage 5 CKD. This exclusion together with ethnic/racial differences in their population may be responsible for the observed difference in the rates. Ninety two percent (92%) of those with LVH per Sokolow-Lyon criteria were less than 60 years of age. The high prevalence of LVH in CKD patients at such young age is rather worrying. This has enormous cardiovascular and socioeconomic implications for the affected persons, their families and the country as a whole. This also further worsens the burden on the health system and the few nephrologists who work in it. The presence of LVH and other CKD related complications like anaemia, hypertension and fluid overload adversely impacts cardiovascular morbidity and mortality in CKD patients [2–4].

There was a positive correlation between LVH and SBP, DBP as well as pulse pressure. The very high prevalence of LVH among the respondents at such early age appear to be related to late presentation and poor control of blood pressure, as LVH is a recognized evidence of end organ damage from uncontrolled hypertension. Among all study participants, the mean systolic and diastolic blood pressures of 167.9 ± 39.8 and 101.8 ± 24.4 respectively are rather high. High blood pressure potentiates the decline in GFR in patients with established diabetic and non - diabetic kidney disease [27]. On the other hand, the development of CKD is a cause of secondary hypertension and can worsen pre-existing hypertension increasing the incidence of resistant hypertension [28]. Hypertension as a CKD complication is an important risk factor for left ventricular hypertrophy. It is therefore essential to adequately treat hypertension to forestall the decline in GFR and limit its impact on the development of LVH that results from poor blood pressure control. High blood pressure remains a major challenge to health care systems in both developed and developing countries [23]. The scale of the problem mandates investment in primary and secondary healthcare as well as patient education.

It is also important to emphasise utilisation of antihypertensive agents that can result in regression or even prevent development of LVH. Attainment of LVH regression is desirable as it reduces adverse outcomes like cardiac arrhythmias and sudden death [29, 30]. Left ventricular hypertrophy was associated with both high systolic and diastolic blood pressures. This is in agreement with other published data [11, 22, 23, 31]. There were gender differences in the prevalence of LVH as the majority of respondents were males; this is in accord with findings from other studies [14]. This may be due to gender differences in body size as left ventricular mass is a function of body size. In the present study increased body mass index was significantly associated with LVH (OR 1.9 CI 1.4-3.7, p = 0.04). The finding that male gender is a determinant of LVH is similar to the findings from studies from developing [14, 16] and developed countries [17]. There was no association between anaemia and LVH. This is in contrast to the findings of Chijioke et al [13] and other studies from the western world [11, 21, 32] in which anaemia has been found to be a determinant of LVH. Proteinuria was not associated with LVH in this study. There appears not to be a consistent association between the LVH and proteinuria; while some studies [32, 33] report proteinuria to be a determinant of LVH, other studies found no such association [17]. Indeed McQuarie et al [17] reported that the association between LVH and proteinuria is lost in patients with stage 3-5 CKD. Our study population comprised exclusively stage 3-5 patients and this probably accounts for the similar findings in the present study.

No echocardiographic assessment of LVH was done in our study, so we were unable to determine the diagnostic test performance of ECG LVH in this population. Although our sample size was moderately large, we may have missed relationships that may have been significant if the strength of the relationship was not high. Finally, ambulatory Blood pressure monitoring would have been a preferred means of confirming high blood pressure.

## Conclusion

In conclusion, there is a high prevalence of left ventricular hypertrophy among CKD patients in Kumasi, Ghana. High diastolic blood pressure, high pulse pressure and increased BMI as well as male gender were found to be significantly associated with the presence of LVH in this cohort. Adequate control of blood pressure is important to forestall the development of CKD and other end organ damage. Early detection of LVH and interventions aimed at prevention and/or regression of LVH are to be encouraged.

### What is known about this topic

- CKD patients are at significantly increased risk for both morbidity and mortality from cardiovascular disease;
- There is data from the developed world on the incidence of left ventricular hypertrophy and its impact on cardiovascular outcomes in CKD patients.

### What this study adds

- To the best of our knowledge, this study is the first report of the high burden of LVH in Ghanaian CKD patients;
- Our study found pulse pressure, high DBP, increased BMI and male gender to be significant associated factors of LVH in our Ghanaian CKD cohort.

## Competing interests

The authors declare no competing interest.
